# Investigating the histological and structural properties of tendon gel as an artificial biomaterial using the film model method in rabbits

**DOI:** 10.1186/s40634-021-00434-y

**Published:** 2022-01-03

**Authors:** Kengo Shimozaki, Junsuke Nakase, Yoshinori Ohashi, Toru Kuzumaki, Tatsuya Yamaguchi, Kojun Torigoe, Hiroyuki Tsuchiya

**Affiliations:** 1 Department of Orthopaedic Surgery, Graduate School of Medical Sciences, Kanazawa University, 13-1 Takara-machi, Kanazawa-shi, Ishikawa-ken, 920-8641 Japan; 2 Department of Materials Science School of Engineering, Tokai University, 4-1-1 Kitakaname, Hiratsuka-shi, Kanagawa-ken, Tokyo, 259-1292 Japan; 3 Department of Rehabilitation, Faculty of Health Science, Fukui Health Science University, 55-13-1 Egami, Fukui-shi, Fukui-ken, Japan

**Keywords:** film model method, traction force, intrinsic tendon regeneration, tendon gel, artificial biomaterial

## Abstract

**Purpose:**

This study aimed to evaluate the properties of tendon gel by investigating the histological and structural differences among tendon gels under different preservation periods using a rabbit model.

**Methods:**

Forty mature female rabbits were divided into four groups, each containing ten rabbits, on the basis of *in-vivo* preservation periods of tendon gels (3, 5, 10, and 15 days). We created the Achilles tendon rupture models using the film model method to obtain tendon gels. Tensile stress was applied to the tendon gel to promote maturation. Histological and structural evaluations of the tendon gel were performed before and after applying the tensile force, and the results obtained from the four groups were compared.

**Results:**

Although the day-3 and day-5 tendon gels before applying tensile stress were histologically more immature than the day-10 and day-15 gels, type I collagen fibers equivalent to those of normal tendons were observed in all groups after the tensile process. Based on the surface and molecular structural evaluations, the day-3 tendon gels after the tensile process were molecularly cross-linked, and thick collagen fibers similar to those present in normal tendons were observed. Structural maturation observed in the day-3 tendon gels caused by traction was hardly observed in the day-5, -10, and -15 tendon gels.

**Conclusions:**

The day-3 tendon gel had the highest regenerative potential to become a normal tendon by applying a traction force.

**Supplementary Information:**

The online version contains supplementary material available at 10.1186/s40634-021-00434-y.

## Background

Tendon regeneration following an injury involves intrinsic and extrinsic regenerative processes *in vivo* [18]; more specifically, during the extrinsic regenerative process, neovessels and inflammatory cells invade the surrounding tissues, and fibroblasts are ultimately recruited to continue this regenerative mechanism [10]. However, in the case of surgical repair, the strength of the tendon decreases as it regenerates from scar tissue—which is composed of fibroblasts—through an extrinsic regenerative process [19, 36]. Thus, to solve this problem, animal-based and clinical studies [21, 24] using artificial ligaments and/or tendons as augmented materials have been performed, yielding encouraging results [6, 11, 35]. On the other hand, complications such as noninfectious effusions, synovitis, and foreign body reactions—often resulting in surgical failure—have been reported during treatment with such materials [11, 16]. In addition to these complications, healing by artificial tendons and/or ligaments is different from normal healing; moreover, it is still not widely used in clinical practice. Therefore, new materials are needed for further development in this field.

Torigoe et al. [31] observed the intrinsic tendon regenerative process by placing the ends of transected murine Achilles tendons between two thin films; this technique, known as the *film model method*, was originally developed for evaluating the regeneration of peripheral nerves [30] as well as for observing tendon intrinsic regeneration. They also reported that a translucent gel-like substance—called *tendon gel*—was secreted from the cut tendon stump and it was a key material for tendon regeneration [31]; in addition, maturation of tendon gel induced by the application of mechanical stress was observed via electron microscopy [31]. Similarly, Ohashi et al. [23] reported that tendon gel matures physiologically and histologically under traction; moreover, an appropriate *in vivo* preservation period is needed for optimal traction-induced tendon gel maturation in murine models. These results support other previous reports showing the importance of applying mechanical force for the regeneration of injured tendons [12, 34]. Therefore, these findings open possibilities for the creation of new artificial tendons of biological origin and/or for promoting mature tendon regeneration using new artificial biomaterials *in vivo*. Clinically, this artificial biomaterial could be used to reinforce tendon components in treating several types of injuries, such as Achilles tendon ruptures and rotator cuff tears, perchance with potentially revolutionary outcomes.

To develop a novel intrinsic-regeneration-based technology, we focused on studying the properties of tendon gel. Only small amounts of the gel can be obtained using the small-sized murine models; therefore, we used rabbits, which are medium-sized animals. This is a deviation from previous studies that used murine models [23, 31]. The use of rabbit models would enable obtaining high amounts of gel samples (using the film model method) and examining the detailed structures of the samples. In addition, it is necessary to show that the tendon gel could be produced using the film model method in medium-sized animals for future clinical applications in humans. The core purpose of this study was to determine suitable *in vivo* preservation periods for the treatment of tendon injuries using the film model method in rabbits; the potential of tendon gel as an artificial biomaterial could be evaluated by identifying the best traction timing when the ability of the tendon gel is utilized to the maximum.

## Materials and methods

### Animal experiments

All animal experiments were approved by the Animal Experimental and Use Committee of the Institute for Experimental Animals, Advanced Science Research Center of our institution (approval No. AP-173823), and the experiments were performed according to the animal experiment regulations of the institute.

A total of 40 skeletally mature female Japanese white rabbits (Kitayama Labs, Nagano, Japan, 15–17-week-old) with a mean weight of 3.2 kg (range, 3.0–3.5 kg) were included in the study. The animals were divided into four groups of 10 rabbits each, according to the *in vivo* preservation periods of the film; the preservation periods for the four groups were 3, 5, 10, and 15 days. The sample size was determined based on the ethical restrictions on the number of experimental animals and power analysis performed previously. A power of 0.8 and a significance level of 0.05 for 40 individuals was required for achieving the effect size of 0.5.

As shown below, intrinsic regeneration models of rabbit Achilles tendon were created using the film model method, and tendon gels were obtained thereafter. Then, a tension force was applied to the tendon gel to promote maturation; histological and structural evaluations of the tendon gel were performed before and after the tensile process to understand both the *in vivo* and tension-driven maturation mechanisms.

### Surgical procedure and obtainment of tendon gel

**Fig. 1:**
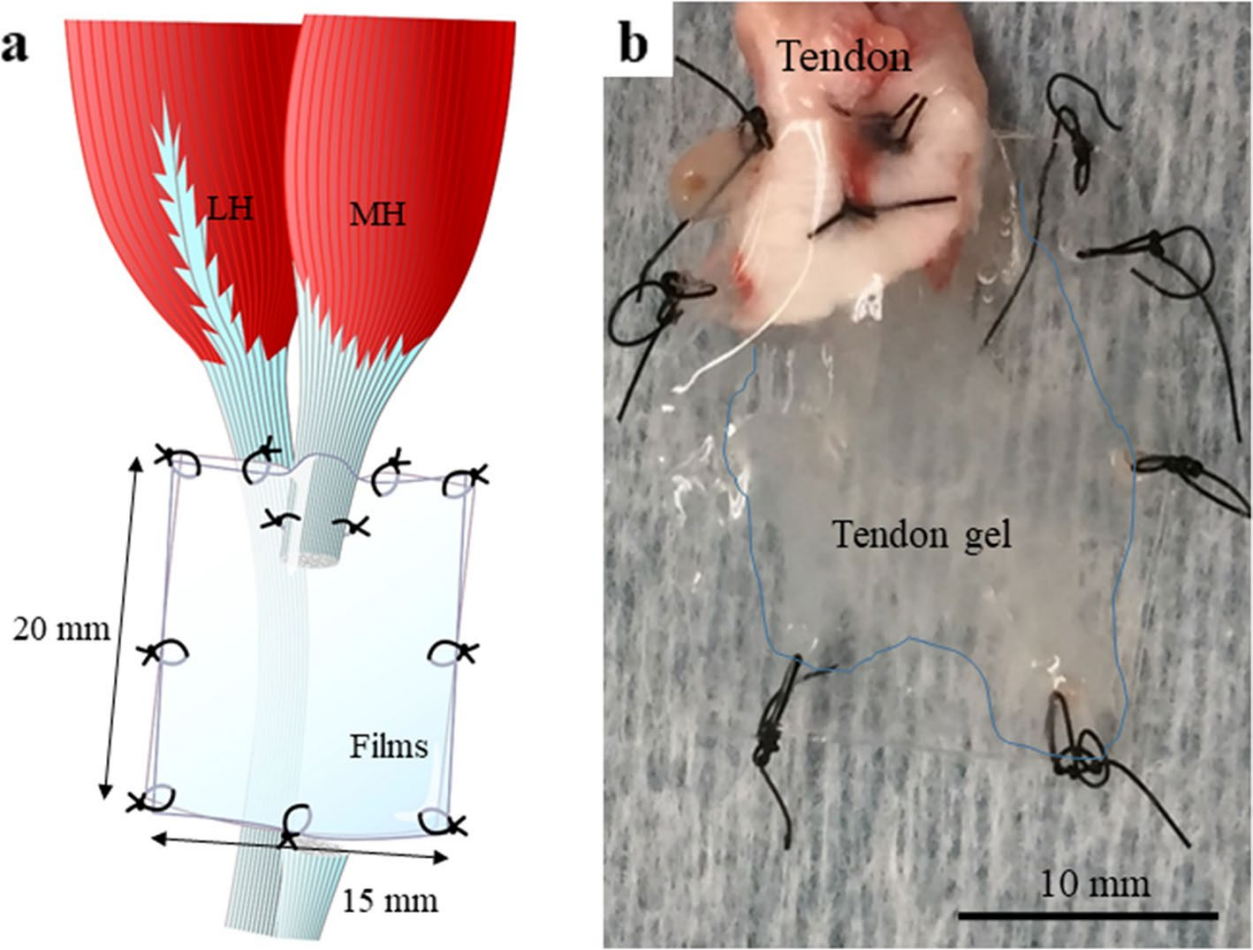
Extraction of tendon gel using film model method. a Film model method using the medial head of the gastrocnemius tendon. b Tendon gel specimen. MH: medial head; LH: lateral head

General anesthesia was induced through preoperative intramuscular injection of a mixture containing 10 mg (2.0 mL) of midazolam and 1.0 mg (1.0 mL) of medetomidine. Subsequently, the fur from the hind limbs, ranging from the gastrocnemius muscle to the Achilles tendon, was shaved; local anesthesia using 30 mg of xylocaine (1.0%, 3.0 mL) per leg was administered, and the surgery was performed using aseptic techniques. Each animal was placed in a prone position on the operating table; in order to create an Achilles tendon rupture model according to the film model method, we bilaterally transected part of the Achilles tendon close to its insertion to the calcaneus on the medial head of the gastrocnemius. After the gastrocnemius muscle belly was eliminated, the synovium, including the paratenon and the sheath around the tendon [28], was removed. Then the proximal end of the transected tendon was inserted between two thin fluorine films (25 μm, 15 × 20 mm, Aflex 25 N NT, Asahi Glass, Tokyo, Japan) and fixed using 6-0 nylon suture (Keisei Medical Industrial Co., Ltd., Tokyo, Japan) [23, 31]. Since these plastic films did not allow the passage of any molecules through it, extrinsic regenerative factors can be eliminated by sandwiching the tendon within two films. Only the proximal end of the collagen bundle was placed on the film after tenotomy; the operated tendon was then moistened with several drops of Ringer’s solution and covered with another film. The four corners as well as the ends of the films were also fixed using 6-0 nylon sutures with at least eight stitches (Fig 1a). After surgery, no external brace was left on the hind limbs. The wrapped tendon ends were left in the body for 3 (day-3 group: D3), 5 (day-5 group: D5), 10 (day-10 group: D10), and 15 days (day-15 group: D15) after surgery. All the 10 rabbits per group (20 limbs) were euthanized on days 3, 5, 10, and 15 after tendon surgery to extract the tendon gels (Fig 1b); thus obtaining 20 tendon gel samples per group.

### Tensile process for tendon gel

**Fig. 2:**
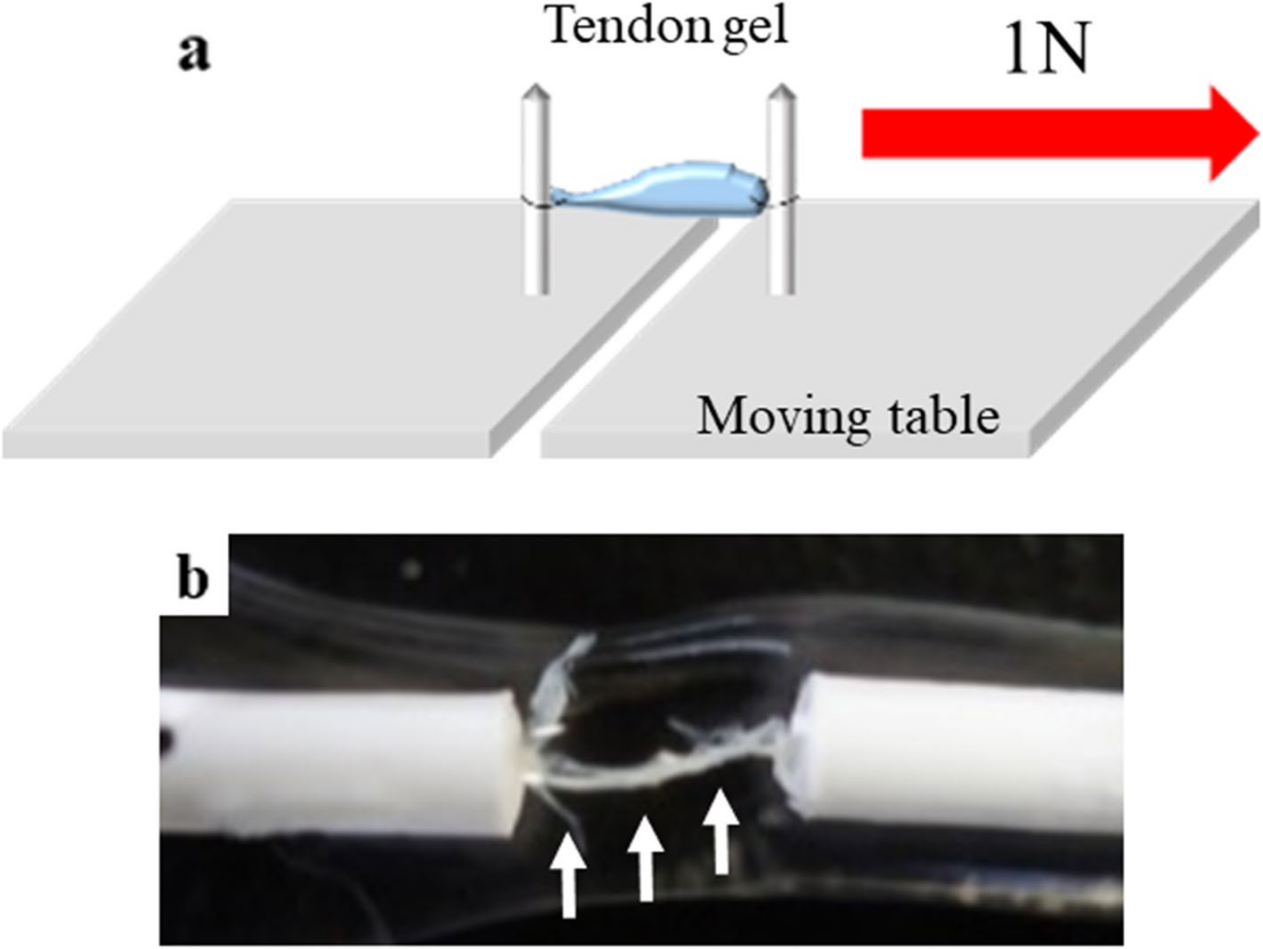
Tensile process for tendon gel. **a** Tensile process unit. **b** Tendon gel after the tensile process (white arrow)

The tensile process was conducted on 10 tendon gel specimens per group. After film removal and separation from the tendon, only the tendon gels were set on the tensile unit, which consisted of an originally designed specimen holder and traction system (Fig 2a). Drops of Ringer’s solution were added onto the tendon gel, and the specimens were placed away from the sunlight, under normal temperature and pressure conditions. The tendon gel specimens were pulled with a sustained 1-N load nearly up to breakage (Fig 2b). This process was recorded every 30 min using a digital camera, and it took an average of 30 ± 6.1 h (range: 18–42 h) until tendon gel breakage. Consequently, we obtained 10 tensed tendon gel specimens per group.

### Histological evaluation

The tendon gel specimens were subjected to hematoxylin-eosin (HE) staining, Elastica van Gieson (EVG) staining, and immunohistology staining with type I and III collagen before and after the tensile process. The histological maturation of the tendon gels before the tensile process was assessed using a semi-quantifying original scoring system based on the Bonar scale with HE and EVG staining [7, 17], which consisted of four parameters evaluated with a maximum of eight points.

The following parameters of the tendon gels before and after the tensile process were examined using a light microscope (KEYENCE BZ-900, Keyence, Osaka, Japan) at 200× magnification: (1) cell count ratio between the tendon parenchyma/tendon gel junction (J) and the central part of the tendon gel (C), (2) ratio between round and flat cells in C, (3) number of connecting junctions between the tendon parenchyma and tendon gel, and (4) amount of collagen fibers. Parameters (1), (2), and (3) were evaluated using HE staining, and parameter (4) was evaluated using EVG staining. Red regions using the EVG stain suggested the presence of collagen fibers. These regions become more apparent as the amount of collagen fiber increases. These parameters were rated using numerical scores that were used as indices of tendon maturity: (1), 0–1 point; (2), 0–2 points; (3), 0–2 points; and (4), 0–3 points (Table 1). However, as the tendon gels after the tensile process had no connecting junctions between the tendon parenchyma and tendon gel and as it was difficult to confirm the ratio of the positions and shapes of the cells, the numerical scoring system was not suitable for use. For the immunostaining, formalin-fixed, paraffin-embedded tissue sections were stained using a standard protocol as described previously [38]. Goat anti-collagen I (goat IgG, cat. no. 1310-01; Southern Biotech) and mouse monoclonal anti-collagen III (mouse IgG_1_ (clone FH-7A), cat. no. ab6310; Abcam) were used as primary antibodies, and the sections were counterstained with 4′,6-diamidino-2-phenylindole (DAPI) for nuclear staining. The confocal imaging was carried out using an inverted Nikon Eclipse Ti2 confocal microscope (Nikon Instruments/Nikon Corp., Tokyo, Japan) equipped with an Andor Dragonfly spinning-disk unit, Andor EMCCD camera (iXon DU888; Andor Technology Ltd., Oxford Instruments), and a laser unit (Coherent Inc., Santa Clara, CA, USA). Excitation for the DAPI and Alexa 488 chromophores was achieved using a 405- and 488-nm laser, respectively. The presence of type I and III collagen fibers before and after the tensile process was evaluated. In this evaluation of both type I and III collagen fibers, green with high intensity, as observed in the normal tendon with type 1 collagen staining, was positive for collagen staining, whereas green with weak intensity was considered to be an artifact and evaluated as negative. Blue indicated the nucleus.

**Table 1:** Histological maturity scores of the tendon gels before the tensile process (0–8 points)

	Score 0	Score 1	Score 2	Score 3
Cell count ratio (J/C)	> 1	≤ 1		
Flat-cell-to-round-cell ratio	0	< 1	≥ 1	
Number of connecting junctions between tendon parenchyma and tendon gel	0	1	2	
Amount of collagen fibers	No strain	Mild strain	Moderate strain	Equivalent to normal tendon

The observation sites were randomly selected from the center and the tendon parenchyma/tendon gel junction of tendon gel specimens, which were independently evaluated by two examiners. The maturity scores were defined as the mean values of the scores determined by the two examiners.

### Structural evaluation

The surface structures of specimens—particularly collagen fibers—were evaluated using atomic force microscopy (AFM) (SPM-9700, SHIMAZDU, Japan). The thickness of the collagen fibers in the four groups was evaluated and compared. A cantilever was used for surface observations in the tapping mode. Molecular structural analysis was carried out using microscopic Fourier transform infrared (FT-IR)

(IRT5000, FT/IR4200A, JASCO, Japan) spectroscopy. This method can detect the presence and amount of cross-linked collagen fibers using the peak of the fatty acid aldehyde group (-CHO), as reported by Kuzumaki et al. [14]. In FT-IR analysis, the aperture diameter was 50 × 50 μm and the integration count was 40. We also evaluated the surface collagen fibers and molecular structures of the tendon gel specimens in each group before and after the tensile process as well as the normal collagen fibers of rabbit Achilles tendons. The specimens for the AFM observations and FT-IR measurements were placed on a moist glass substrate. We tried not to damage the tendon gels during this placement to avoid affecting the experimental results.

## Statistical analysis

The data were analyzed using the Statistical Package for the Social Sciences for Windows (version 23.0; IBM, Armonk, NY, USA). ANOVA was performed on the histological evaluation scores and the thickness of the collagen fibers in the four groups, and statistically significant (i.e., *p* <0.05) items were examined using a *post hoc* multiple comparison test (i.e., Dunnett test).

## Results

**Fig. 3:**
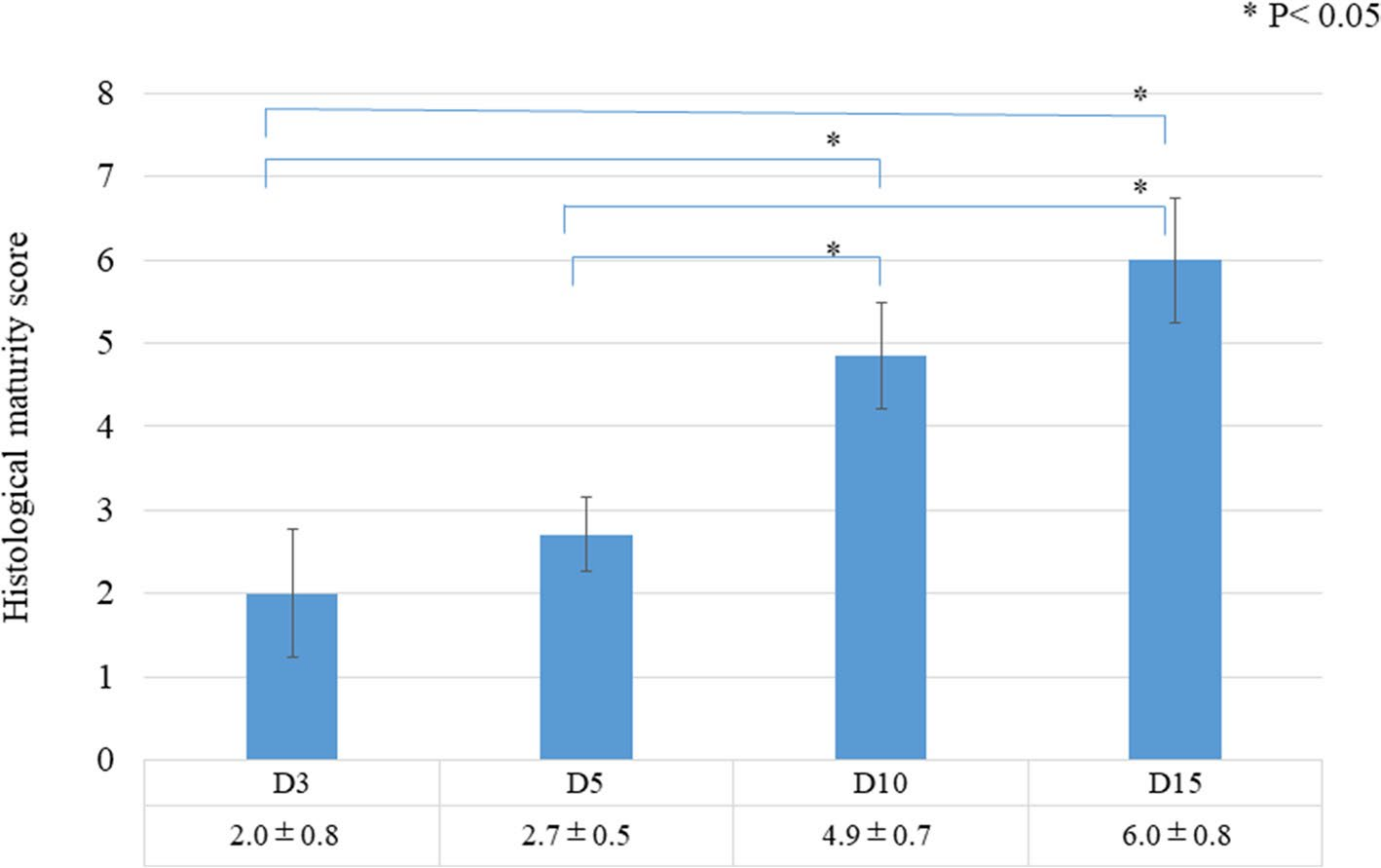
Multiple comparison tests concerning the histological assessment before the tensile process

**Fig. 4:**
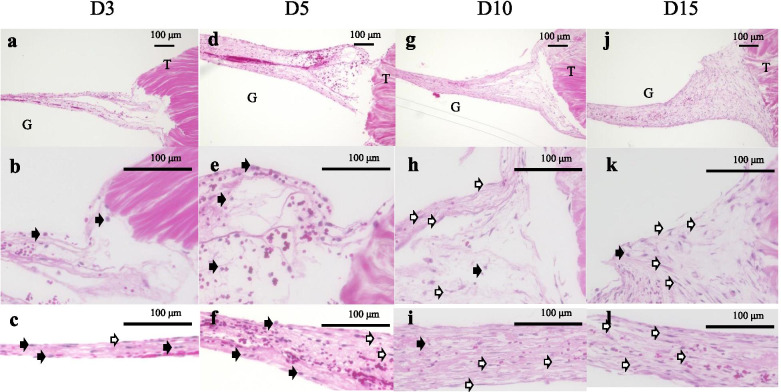
Evaluation of hematoxylin-eosin staining in all groups. (**a**), (**d**), (**g**), and (**j**): View of tendon gels in the low-power field at 40× magnification. (**b**), (**e**), (**h**), and (**k**): Magnified view of the junction between tendon and tendon gel at 200× magnification. (**c**), (**f**), (**i**), and (**l**): Magnified view of the central part of tendon gel at 200× magnification. White arrow: flat cells; black arrow: round cells; T: tendon; G: tendon gel

**Fig. 5:**
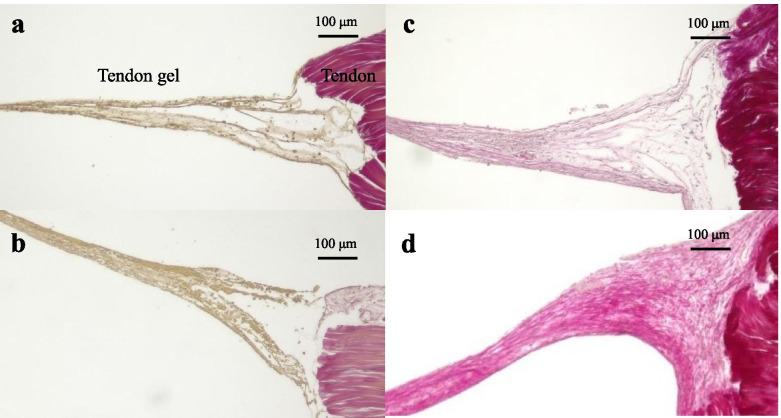
Evaluation of Elastica van Gieson staining in all groups. **a** D3 and **b** D5 were not stained; **c** D10 was mildly stained; and **d** D15 was moderately stained

**Fig. 6:**
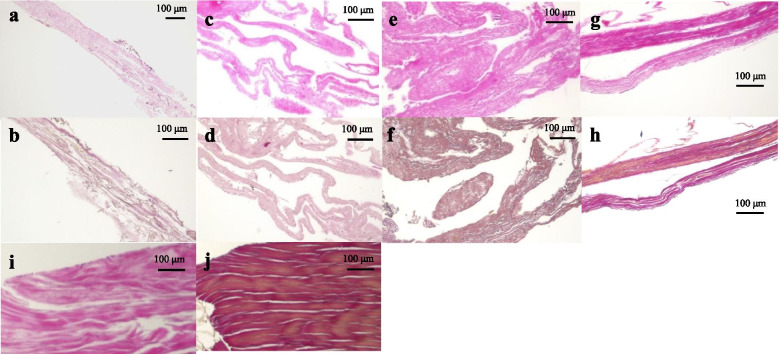
Histological evaluation after the tensile process. **a** HE and **b** EVG staining of D3; **c** HE and **d** EVG staining of D5; **e** HE and **f** EVG staining of D10; and **g** HE and **h** EVG staining of D15. The amount of connective tissue fibers increased in HE staining, and all tendon gels following the tensile process were EVG-stained equivalently to a normal tendon. Normal tendon example in **i** HE stain and **j** EVG stain. HE: hematoxylin-eosin, EVG: Elastica van Gieson

**Fig. 7:**
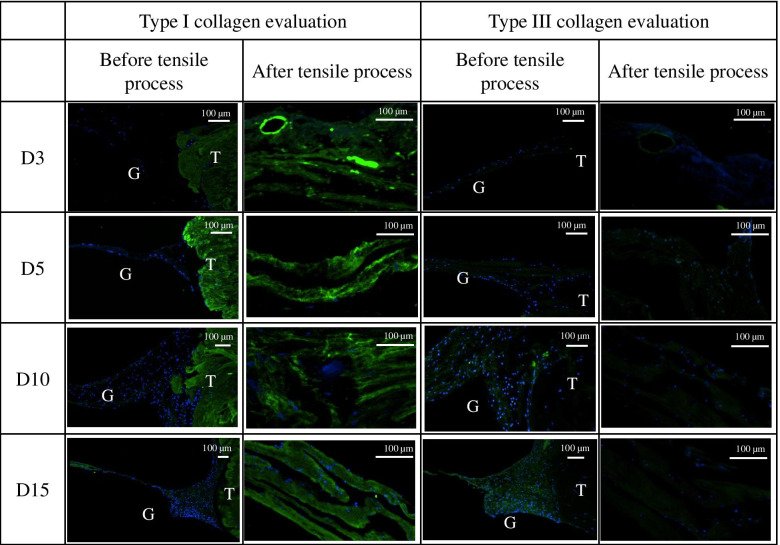
Immunohistological evaluation of type I and III collagen before and after the tensile process. In both staining methods, the presence of each collagen type is indicated by the green area. The presence of type I collagen was validated for all the groups after the tensile process, although it was confirmed only in D15 before the tensile process. Similar to the type I collagen staining, the presence of type III collagen was confirmed in D10 and D15 prior to the tensile process. There were barely any type 3 collagen fibers in any of the groups after the tensile process. Green color: the area where collagen is present; Blue color: nucleus; T: tendon; G: tendon gel

**Fig. 8:**
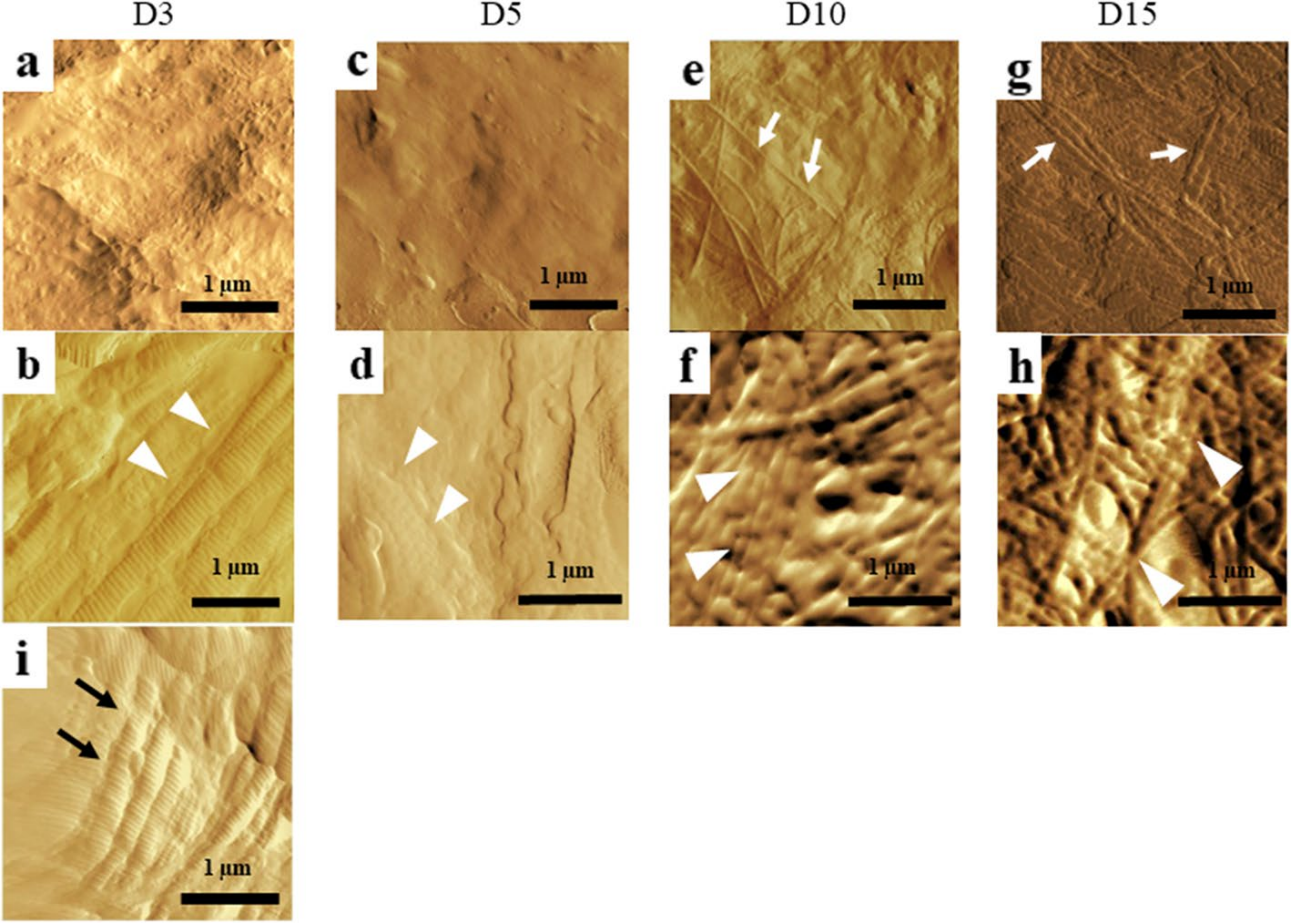
Surface analysis using atomic force microscopy in each group. There were almost no collagen fibers in (**a**) D3 and (**c**) D5 before the tensile process. In (**e**) D10 and (**g**) D15 prior to the tensile process, collagen fibers (white arrows) were identified, albeit thinner than those in normal tendons (i; black arrow). In contrast, the arrangement of the collagen fibers equivalent to that of normal tendons (white arrowhead) was demonstrated in (**b**) D3 and (**d**) D5 after the tensile process. The arrangement of the collagen fibers was also noted in (**f**) D10 and (**h**) D15, although fibers were thinner than those in (**i**) normal tendons

**Fig. 9:**
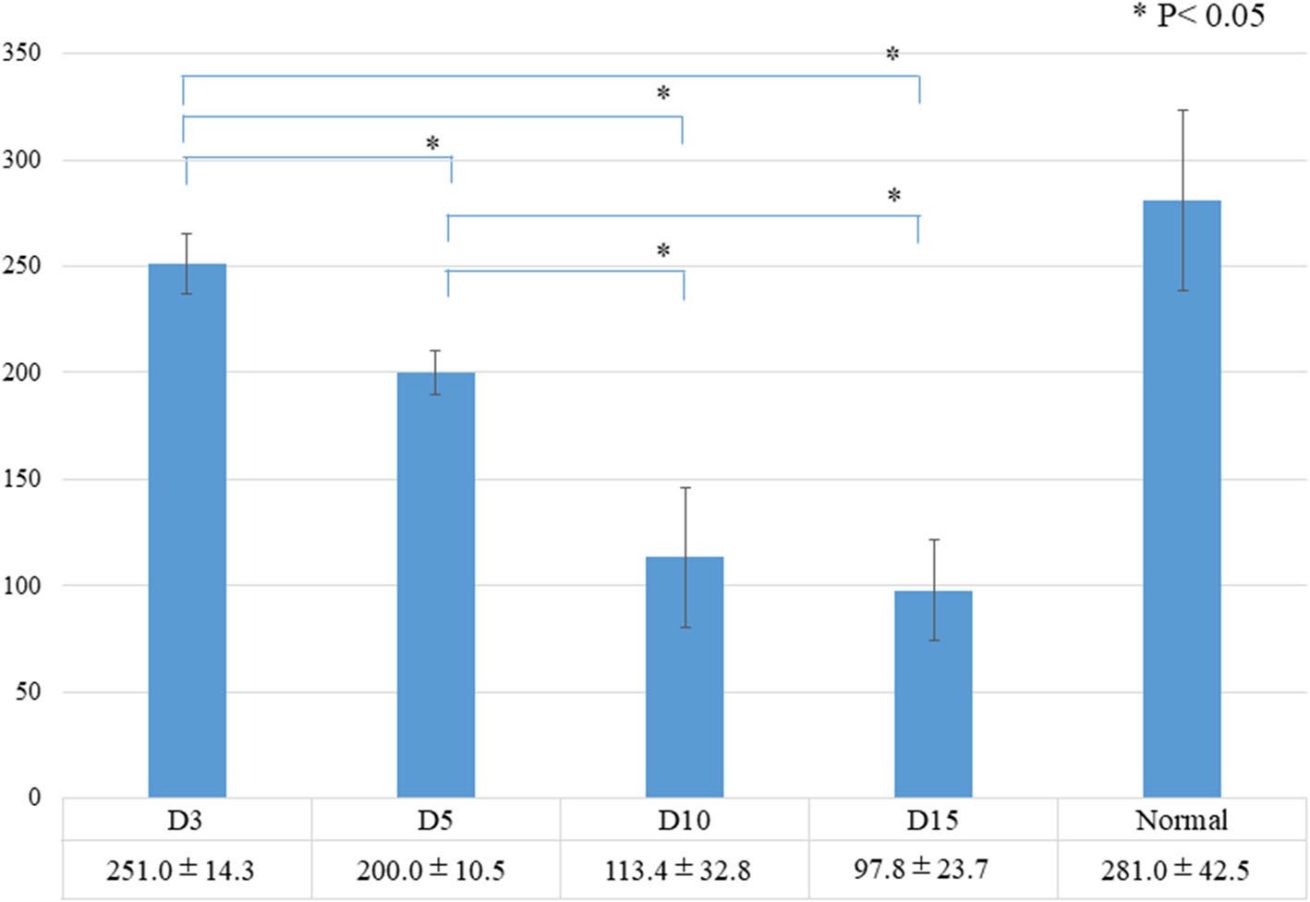
Surface analysis for evaluating the thickness of collagen fibers after the tensile process

**Fig. 10:**
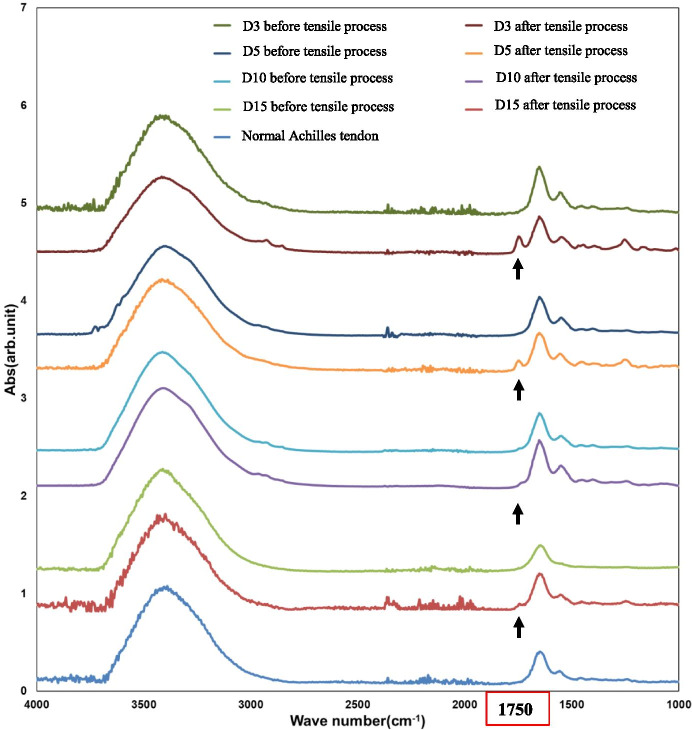
Molecular structural analysis. A -CHO peak close to 1,750 cm-1 indicating cross-linking between collagen fibers was observed in all groups after the tensile process (black arrows), which was not observed before the tensile process and in normal tendons. This peak tended to be higher for D3 than for D5, D10, and D15

Concerning the histology before the tensile process, a significant difference in the total histological evaluation score (0–8) among the four groups was detected using analysis of variance (ANOVA, *p <* 0.01). The *post hoc* multiple comparison test revealed a significant difference between the groups euthanized before D5 and after D10 (D3, 2.0 ± 0.8 vs. D5, 2.7 ± 0.5, *p* = 0.20; D3 vs. D10, 4.9 ± 0.7, *p* < 0.01, D3 vs. D15, 6.0 ± 0.8; *p* < 0.01; D5 vs. D10, *p* < 0.01; D5 vs. D15, *p* < 0.01; D10 vs. D15: *p* = 0.08) (Fig 3). Tendon gel specimens after staining with HE and EVG stains are shown in Figs 4 and 5. The cell nucleus of the tendon gel changed from round to flat, moving inward along with maturation. Additionally, the gels turned red over time with EVG staining. The red color in the EVG stain suggests the presence of collagen fibers, with the color intensity increasing with the amount of collagen fibers. No invasion by new blood vessels was observed in any tendon gel. After the tensile process, collagen fibers arranged similarly to those in a normal tendon were observed in all the groups (Fig 6). In the immunohistological evaluation, type I collagen fibers in the tendon gel were demonstrated to exist in D15 before the tensile process although only the normal tendon was stained in the other groups (Fig 7). In contrast, type I collagen fiber was observed in every group after the tensile process. Type III collagen fibers in the tendon gel were verified to exist in D10 and D15 before the tensile process but were barely observed in any of the groups after the tensile process. This result indicated that the tendon gels of all the groups had matured into collagen fibers composed mainly of type I collagen as a result of the tensile process. The AFM surface analysis results showed that there were almost no collagen fibers in D3 and D5 before the tensile process. However, in D10 and D15 before the tensile process, collagen fibers were thinner than those in normal tendons (D10: 51.4 ± 3.6 μm × 10^-3^; D15: 87.2 ± 3.9 μm × 10^-3^; Normal tendon: 281.0 ± 3.9 μm × 10^-3^). Moreover, collagen fibers in D3 after the tensile process were arranged as in normal tendons; in contrast, collagen fibers in D5, D10, and D15 were still thinner than those in normal tendons (Fig 8). Furthermore, a significant difference in the thicknesses of collagen fibers after the tensile process was detected between D3/D5 and D10/D15, with D3 and D5 having thicker fibers. In addition, the collagen fibers in D3 were significantly thicker than those in D5. However, there was no significant difference between the collagen fibers in D3 and those in normal tendons (Fig 9). According to molecular structural analysis results, a -CHO peak close to 1,750 cm^-1^ indicating the occurrence of cross-linking of collagen fibers was observed in all groups after the tensile process, which was not observed either before the tensile process or in the normal tendons (Fig 10). In addition, this peak tended to be larger in D3 than in D5, D10, and D15.

## Discussion

This study showed that D3 gel exhibits higher regeneration-promoting ability before the tensile process than that of D5, D10, and D15 gels. Various intermolecular cross-links occurred in D3 gel because of the tensile process; therefore, histologically and structurally mature gels were observed. The tendon gel was matured by muscle contraction after the formation of junction between the tendon parenchyma and tendon gel from the fifth day. Surface and molecular analyses indicated that the histologically immature D3 tendon gel before the tensile process was matured because of the traction force, unlike D5, D10, and D15 gels, indicating the high potential of D3 gel as a novel biomaterial for applications in regenerative medicine.

Histologically immature tendon gels, such as D3, were considered to belong to a premature stage. Unlike D10 and D15 gels, histological and structural maturity could be achieved in the immature gels through traction force. Collagen fibers in tendons are organized hierarchically [26]; therefore, intermolecular cross-linking could occur along the traction direction during the tensile process. The D3 tendon gels contained numerous tropocollagens before the tensile process. The collagen molecules in D10 and D15 tendon gels were cross-linked in multiple directions because multidirectional traction forces were exerted by muscle contraction *in vivo.* Therefore, the number of cross-links after the tensile process was less and the collagen fibers were thin. D5 gels were histologically immature before the tensile process, and their collagen fibers were thinner than those of normal tendons; structural analysis results revealed a significant difference between D3 and D5 gels after the tensile process. D5 gel was more mature than D3 gel *in vivo*. Therefore, D3 gels before the tensile process were more suitable than D5 gels for maturation using traction force; furthermore, D3 gels showed enhanced regenerative potential, making them suitable for use as artificial biomaterials.

Tendons generally contain a few flat tenocytes [3, 9]. However, D3 and D5 gels contained many round tenocytes. Ohashi et al. reported that [23] tenocytes in the murine models were generated at the tendon edges during the early stage, spreading throughout the gel, and their shapes changed from round to flat, indicating *in vivo* maturation. We observed a histologically significant difference between D3/D5 and D10/D15 gels before the tensile process, which was because of the completion of connecting junctions between the tendon parenchyma and tendon gel. In D10/15 gels, which comprised connecting junctions, the change in cell morphology from round to flat could be attributed to cell maturation *in vivo* owing to the traction stress exerted by the gastrocnemius.

Collagen fibers are organized in hierarchical levels in tendon tissue such as tropocollagen—a triple-helix peptide chain in fibrils, fascicles, tertiary bundles, and the whole tendon [1, 26]. When the extrinsic and intrinsic regeneration processes are mixed, as observed in the clinical scenarios, the tenocytes and collagen fibers are aligned in the direction of mechanical stress with concurrent decreases in type III collagen and water content in scar tissue formation [36]. The increased collagenase activity eventually supports type III collagen resorption and its subsequent replacement with type I collagen gradually [10, 25]. Our results excluding the extrinsic factors showed that the tissue composed of type I collagen, similar to the normal tendon, is regenerated in a short time by applying a traction force to the tendon gel produced by intrinsic regeneration. Immunohistological evaluation showed type I collagen only in D15 and type III collagen in D10 and D15 before the tensile process and no collagen fiber in D3 and D5. This fact is supported by our hypothesis that tendon gel maturation is gradually achieved after the formation of the junction between the tendon parenchyma and tendon gel.

The presence of peaks close to 1,750 cm^-1^ was confirmed in all four groups after the tensile process during the molecular structural evaluation. The D3 peak was higher than D5, D10, and D15 peaks. The FT-IR spectrum analysis reported the first cross-linking reaction as *aldol condensation,* where a saturated -CHO group was formed [2, 27, 29], which resulted in a peak closer to 1,750 cm^-1^. Peak height relates to the extent of cross-linking. Our results showed no peak in the normal mature tendons [14]. A previous study [33] reported that the structural stability of collagen fibers is affected mostly by the intermolecular cross-links. The collagen fibers are more strongly bound together when more fibers are cross-linked in type I collagen. Mechanical stress is essential for collagen fiber maturation [14, 32]. Kwansa et al. [15] reported that traction force promotes cross-linking between the amino- and carboxy-terminal extremities of collagen fibers, which structurally strengthen the entire collagen tissue. Therefore, intermolecular cross-links occurred more frequently in D3 than in the other groups.

Thick collagen fibers similar to those in normal tendons were identified in D3 after the tensile process by AFM surface analysis. The intermolecular cross-links formed in collagen after traction force was considered to cause collagen maturation. The presence of thick collagen fibers, equivalent to those in normal tendons, was confirmed on tendon gel surface. In D5, D10, and D15, as the -CHO peak was small, the number of intermolecular cross-links was also considered small because thick collagen fibers were not detected during surface analysis. D3 gels before the tensile process can form collagen intermolecular cross-links and thick collagen fibers through the traction force and subsequently undergo maturation.

In a preliminary study, day-1 and day-2 tendon gel models (three tendon gel samples for each day) were also prepared; however, no tendon gel of appreciable size was obtained.

An advantage of this study was that the animal experiments were conducted using the film model method. With the careful removal of paratenon and synovial tissues, this method eliminated the extrinsic regeneration activity, which inhibits intrinsic regeneration. The possibility of regenerating tendons histologically and structurally similar to those before an injury was verified by applying a traction force on the early-stage D3 tendon gel. We believe that our findings can lead to two possible clinical scenarios: first, if an *in-vivo* environment capable of removing the extrinsic regeneration component is created in future, using the inhibitory factors of the vascular endothelial growth factor [4, 20] or some inflammatory factors [5], then the tendons similar to those before injury could be regenerated. Second, the early-stage tendon gel could potentially be used as an artificial biomaterial because it could form an artificial tendon through the traction force. Owing to the characteristics of the matured tendon gel, we believe that new therapeutic options could be added to other treatments for tendon injuries, such stem cells, growth factors, collagen fiber scaffolds, and/or gene transfer [8, 13, 22, 38]. The performance of the early-stage tendon gel as an artificial biomaterial should be verified through clinical research. Our study provides useful basic knowledge for scar-free intrinsic tendon regenerative processes.

Study limitations include the lower limbs were not immobilized after the surgery and the tendon gel matured because of the activity of the rabbit. However, as the variation in the histological maturity score of tendon gels in each group was small, the results might not be considerably affected by this limitation. The stressing technique was based on a constant unidirectional traction force; hence, actual muscle contraction consisting of cyclic movement patterns could not be reproduced. Tenocytes related to intrinsic healing were not specifically stained, proving that the tendon gel cells were not tenocytes. As the paratenon was removed and no new blood vessels were observed in the tendon gel, tenocytes might be present in the tendon gel, but needs verification. No statistical comparison among the four groups after the tensile process was performed for histological evaluation, as the tendon gel after the tensile process was separated from the tendon. Therefore, the preparation of tissue specimens that were oriented in the fiber direction and the evaluation of the C and J area using the scoring system was difficult. However, tendon gels after the tensile process uniformly changed according to the histological evaluation and no significant differences were observed. Issues related to the generation period of tendon gels in human tendons and the methodology for creating an *in-vivo* intrinsic-healing-only environment must be addressed in future. In future studies, we intend to use animal bioreactors for producing tendon gels. Collagen has low antigenicity in different species; therefore, artificial material transplantation is relatively easy and feasible. In the present study, the amount of tendon gel obtained from rabbits was sufficient. Therefore, artificial regeneration of substances from rabbits would be possible in the future using technologies that enable increasing the production of tendon gels.

## Conclusions

The histological and structural properties of tendon gels were evaluated for groups with different preservation periods. The day-3 tendon gel had the highest regenerative potential to become a normal tendon by applying a traction force.

## Supplementary Information

**Additional file 1.**

## Data Availability

The datasets generated during and/or analyzed during this study are available from the corresponding author on reasonable request.

## Abbreviations

HE: Hematoxylin-eosin
EVG: Elastica van Gieson
ANOVA: Analysis of variance
DAPI: 4′,6-diamidino-2-phenylindole
AFM: Atomic force microscopy
FT-IR: Fourier transform infrared
