# Supplementary figures and images for: Investigating the histological and structural properties of tendon gel as an artificial biomaterial using the film model method in rabbits
Source: J Exp Orthop. 2022 Jan 3;9:1. doi: 10.1186/s40634-021-00434-y (PMC8724385; doi:10.1186/s40634-021-00434-y)

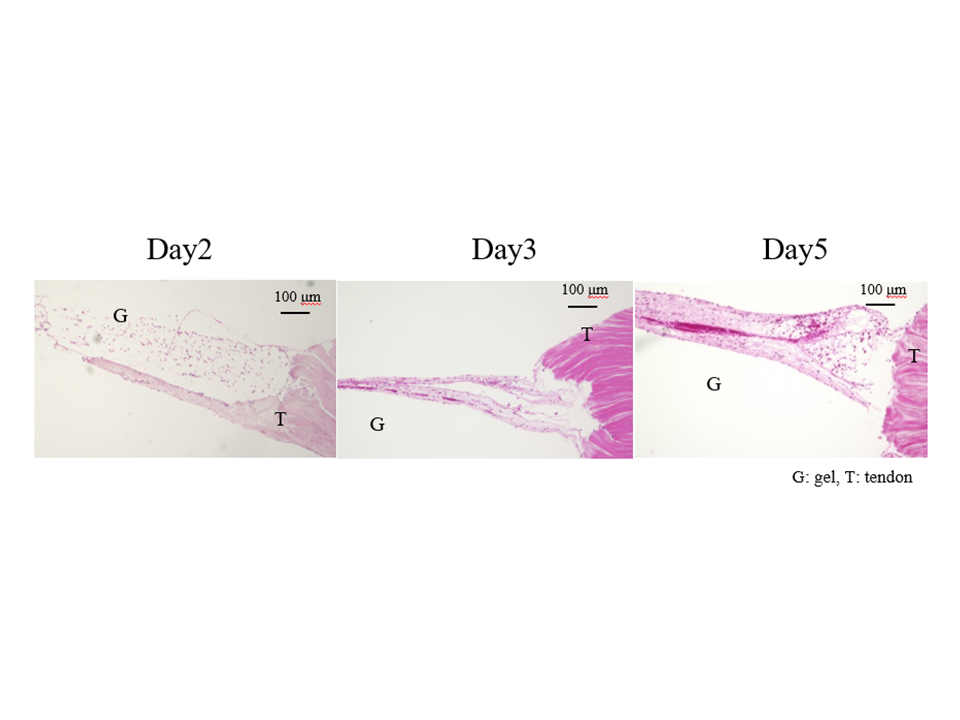

Supplement: Supplementary file 1 — Additional file 1. [file 40634_2021_434_MOESM1_ESM.tif]
